# Antioxidant and Anti-Inflammatory Effects in RAW264.7 Macrophages of Malvidin, a Major Red Wine Polyphenol

**DOI:** 10.1371/journal.pone.0065355

**Published:** 2013-06-05

**Authors:** Eszter Bognar, Zsolt Sarszegi, Aliz Szabo, Balazs Debreceni, Nikoletta Kalman, Zsuzsanna Tucsek, Balazs Sumegi, Ferenc Gallyas

**Affiliations:** 1 Departments of Biochemistry and Medical Chemistry, University of Pecs Medical School, Pecs, Hungary; 2 Heart Institute, University of Pecs Medical School, Pecs, Hungary; 3 Nuclear-Mitochondrial Interactions Research Group, Hungarian Academy of Sciences, Budapest, Hungary; 4 Szentagothai Research Center, Pecs, Hungary; Virginia Commonwealth University, United States of America

## Abstract

**Background:**

Red wine polyphenols can prevent cardiovascular and inflammatory diseases. Resveratrol, the most extensively studied constituent, is unlikely to solely account for these beneficial effects because of its rather low abundance and bioavailability. Malvidin is far the most abundant polyphenol in red wine; however, very limited data are available about its effect on inflammatory processes and kinase signaling pathways.

**Methods & Findings:**

The present study was carried out by using RAW 264.7 macrophages stimulated by bacterial lipopolysaccharide in the presence and absence of malvidin. From the cells, activation of nuclear factor-kappaB, mitogen-activated protein kinase, protein kinase B/Akt and poly ADP-ribose polymerase, reactive oxygen species production, mitogen-activated protein kinase phosphatase-1 expression and mitochondrial depolarization were determined. We found that malvidin attenuated lipopolysaccharide-induced nuclear factor-kappaB, poly ADP-ribose polymerase and mitogen-activated protein kinase activation, reactive oxygen species production and mitochondrial depolarization, while upregulated the compensatory processes; mitogen-activated protein kinase phosphatase-1 expression and Akt activation.

**Conclusions:**

These effects of malvidin may explain the previous findings and at least partially account for the positive effects of moderate red wine consumption on inflammation-mediated chronic maladies such as obesity, diabetes, hypertension and cardiovascular disease.

## Introduction

Malvidin and its glycosides are primary plant pigments playing an important role to protect plants from microbial infection and UV irradiation [1]. Malvidin is responsible primarily for the color, and is included in the polyphenols of red wine together with other anthocyanidins, phenolic acids, flavonoids and trihydroxy stilbenes [2], [3]. Recent findings indicate a potential preventive role of dietary polyphenols against chronic inflammatory diseases such as diabetes, hypertension and cardiovascular disease [4]–[7].

The inflammatory response was extensively studied in lipopolysaccharide (LPS)-stimulated RAW 264.7 macrophage cells, which are very sensitive to LPS stimulation and respond by activation of the pro-inflammatory transcription factors; nuclear factor-kappaB (NF-κB) and activator protein-1 (AP-1) resulting in tumor necrosis factor-alpha, interleukin-1beta (IL-1β), IL-6, IL-8 and nitric oxide production [8]–[10]. These markers are associated with gram-negative sepsis and other inflammatory diseases [11]. Furthermore, LPS also induces production of reactive oxygen species (ROS) and activation of the nuclear enzyme poly ADP-ribose polymerase (PARP) [12], [13]. ROS are capable of eliciting a variety of pathological changes, including peroxidation of lipids, proteins, and DNA, and, as a signaling loop, an elevated level of ROS activates mitogen activated protein kinase (MAPKs) and inflammatory transcription factors [14]–[16]. Probably as compensatory mechanisms, LPS induces activation of the cytoprotective phosphytidylinositol 3-kinase (PI3K)-Akt pathway [17] and expression of MAPK phosphates (MKP)-1 [15]. All these processes have significant role in innate immunity during the normal immune response and in causing multiple organ failure and death during severe sepsis or septic shock [11].

The most investigated nutritional polyphenol, resveratrol was found to prolong lifespan, and was suggested as a potential anti-inflammatory, anti-aging, anti-cancer and anti-cardiovascular disease agent [18]-[20]. However, rather low bioavailability and abundance of resveratrol implies that other components may contribute substantially to the beneficial effects of red wine [21], [22]. A likely candidate is malvidin that exceeds resveratrol content at least 100 times in red wines [23]. Recent data describe its beneficial effects in cardiovascular disease [24]. On the other hand, only limited data are available about effect of malvidin on inflammatory processes and kinase signaling pathways [25]–[27]. Therefore, in this study, we investigated the effect of malvidin on LPS induced processes in RAW 264.7 macrophages.

## Materials and Methods

### Chemicals

Bacterial lipopolysaccharide from *Escherichia coli* 0127:B8, trans-resveratrol and Malvidin chloride were purchased from Sigma-Aldrich Co. (Budapest, Hungary). Protease inhibitor mixture was purchased from Sigma-Aldrich Co. (Budapest, Hungary). Antibodies against phosphorylation specific extracellular signal regulated kinase (ERK_1/2_) Thr^183^–Tyr^185^, ERK_1/2_, phosphorylation specific p38 MAPK Thr^180^–Gly–Tyr^182^, p38-MAPK, phosphorylation specific c-Jun N-terminal kinase (JNK), JNK, phosphorylation specific Akt-1/protein kinase B-α Ser^473^, Akt1, phosphorylation specific glycogen synthase kinase (GSK)-3β Ser^9^, NF-κB p65 and phosphorylation specific NF-κB p65(Ser536) were purchased from Cell Signalling Technology, Kvalitex Co. (Budapest, Hungary). Antibody against N-terminal domain of actin was obtained from Sigma-Aldrich Co. (Budapest, Hungary), and MAPK phosphatase-1 (MKP-1), Histon H-1 antibodies were from Santa Cruz Biotechnology (Santa Cruz, CA, USA). Recombinant GSK-3β, c-Jun, myelin basic protein (MBP) and myocyte enhancer factor (Mef)-2 was purchased from Abnova Gmbh (Heidelberg, Germany). JNK Inhibitor II, SB 203580, PD 98059 and Akt Inhibitor IV were from Merck Hungary Ltd. (Budapest, Hungary). Methylthiazolyldiphenyl-tetrazolium bromide (MTT) was purchased from Sigma–Aldrich Co. (Budapest, Hungary). The fluorescent mitochondrial dye 5,5′,6,6′-tetrachloro-1,1′,3,3′-tetraethyl-benzimidazolylcarbocyanine iodide (JC-1) were from Molecular Probes (Leiden, Netherlands). All reagents were of the highest purity commercially available.

### Immunoblot analysis

RAW 264.7 murine macrophage (ECACC, Salisbury, UK) and RAW-Blue™ (Cayla – InvivoGen, Toulouse, France) cells were cultured in 5% CO_2_– 95% air at 37°C in Dulbecco’s Modified Eagle’s Medium (DMEM–endotoxin tested) with 10% fetal calf serum (FCS) and L-glutamine (Sigma-Aldrich, Budapest, Hungary). The cells were seeded at a starting density of 2×10^6^ cells/well to a 6-well plate, cultured overnight then treated or not with 1 µg/ml LPS together or without 0–100 µM malvidin or resveratrol. We pre-incubated RAW 264.7 macrophages in the presence or absence of malvidin or resveratrol for 30 min before the LPS challenge. Cells were harvested in ice-cold lysis buffer containing 0,5 mM sodium metavanadate, 1 mM ethylenediaminetetraacetic acid (EDTA), protease inhibitor mixture and phosphate-buffered saline, pH: 7.4. Proteins were precipitated by trichloroacetic acid, washed three times with –20°C acetone, and subjected to sodium dodecylsulphate (SDS) polyacrylamide gelelectrophoresis. Proteins (30 µg/lane) were separated on 12% gels and then transferred to nitrocellulose membranes. Membranes were blocked in 5% low fat milk for 1 h at room temperature, then exposed to the primary antibodies at 4°C overnight at a dilution of 1:1,000. Appropriate horseradish peroxidase-conjugated secondary antibody was used for 2 h at room temperature in 1∶5000 dilution (Sigma-Aldrich Co, Budapest, Hungary). Peroxidase labeling was visualized with enhanced chemiluminescence using the SuperSignal West Pico chemiluminescent substrate (Pierce Chemical, Rockford, IL, USA). Developed films were scanned, and pixel volumes of the bands were determined using NIH Image J software. All experiments were repeated three times.

### Cell viability assay

Cells were seeded to 96-well plates at a starting density of 2×10^4^ cells/well and cultured overnight. We pre-incubated RAW 264.7 macrophages in the presence or absence of 50 µM malvidin for 30 min, then exposed or not the cells to 1 µg/ml LPS for 24 h. Media were replaced for fresh one without any agentscontaining 0.5% of the water-soluble mitochondrial dye, MTT. Incubation was continued for 3 more hours, and MTT reaction was terminated by adding 1/10 volume of 10% of SDS solution containing 0.1 M HCl. The amount of water-insoluble blue formasan dye formed from MTT was proportional to the number of live cells and was determined using a 96-well plate reader (Anthos Labtech 2010; Vienna, Austria) at 550 nm wavelength after dissolving the blue formasan precipitate in the acidic SDS solution. All experiments were performed in at least four parallels and repeated three times.

### Determination of intracellular reactive oxygen species

Intracellular ROS were determined using the oxidation-sensitive 2,4 dichlorodihydrofluorescein-diacetate (C-400, Invitrogen) fluorescent dye. Cells were seeded into 96-well plates at a starting density of 2×10^4^ cell/well, then cultured overnight. Culturing medium was replaced with a fresh one. RAW 264.7 cells were incubated or not in the presence of 1 µg/ml LPS together with 0–50 µM malvidin or trans-resveratrol for 22 h. Then C400 at a final concentration of 2 µg/ml was added to the medium for an additional 2 h. Fluorescence was measured at 485nm excitation and 555nm emission wavelengths using Fluostar Optima (BMG Labtechnologies) fluorescent microplate reader. All experiments were performed in at least 6 parallels and repeated three times.

### NF-κB activation assay

RAW 264.7 macrophages were transiently co-transfected with either NF-κB luciferase or control (TA-Luc) (Panomics, Santa Clara, CA, USA), and SV-β-galactosidase (pSV-β-gal) (Promega Corporation, Madison, WI, USA) plasmids by using Lipofectamine 2000 transfection reagent according to the manufacturer's instructions. 24 h after the transfection, cells were treated as indicated, and another 24 h later cell lysates were collected. Cellular proteins were assayed for luciferase and β-galactosidase activities according to the manufacturer's instructions (Promega Corporation, Madison, WI, USA, Luciferase Assay System Technical Bulletin TB281). The ratio of luciferase to β-galactosidase activity served to normalize the luciferase activity to correct for any differences in transfection efficiencies.

Alternatively, RAW-Blue™ cells were treated as indicated for 24 h, then the medium was replaced by QUANTI-Blue™ detection medium (Cayla – InvivoGen, Toulouse, France) for 1h. RAW-Blue™ cells are permanently transfected with an NF-κB- and AP-1-sensitive promoter-driven alkaline phosphatase. NF-κB and AP-1 binds to the promoter upon nuclear translocation, and induces the expression of alkaline phosphatase that is detected by a dye-based assay and a plate reader.

### Detecting mitochondrial membrane potential (Δψ)

The changes in Δψ were assayed using the Δψ dependent fluorescent dye, JC-1. RAW 264.7 cells were seeded at 1×10^6^ cells/well starting density to a six-well plate containing coverslips and cultured at least overnight before the experiment. After subjecting the cells to the appropriate treatment (indicated in the figure legends), coverslips were rinsed twice in phosphate buffered saline (PBS). Coverslips were placed face down on top of a microscope slide forming a small chamber filled with PBS supplemented with 0.5% FCS and containing 5 µg/ml JC1 (Molecular Probes). Cells were imaged with a Zeiss Axiovert 25 fluorescent microscope equipped with a ProgRes C12 Plus CCD camera using a 63× objective and epifluorescent illumination. For JC-1 fluorescence, cells were loaded with the dye for 15 min at 37°C, then the same microscopic field was imaged first with 546 nm bandpass excitation and 590 nm emission (green filter, red fluorescence), then with 450–490 nm bandpass excitation and 520 nm emission (blue filter, green fluorescence). Resulting images were merged. In control experiments, we did not observe considerable bleed-through between the red and green channels.

### RNA extraction and quantitative reverse transcriptase polymerase chain reaction (Q-RT-PCR) amplification

Total RNA was extracted from RAW 264.7 cells using TRIZol reagent (Sigma-Aldrich), according to the manufacturer’s protocol. RNA (1 µg) was reverse-transcribed with MMLV RT (RevertAid™ first-strand cDNA synthesis kit, Fermentas, Burlington, Ontario, Canada) for 1h at 42°C; final volume was 20 µl. cDNA (1 µl) was used for real-time PCR amplification on a Bio-Rad Mini Opticon (MJ Mini) machine. PCR was conducted over 45 cycles of 95°C for 15 s, 55°C for 30 s, and 72°C for 45 s; three-step thermal cycling preceded by an initial 95°C for 7 s using the iQ SYBR Green Supermix kit (Bio-Rad, Hercules, CA, USA). PCR was performed using the following primers:

MKP-1 forward, 5′-GCATCCCTGTGGAGGACAACC-3′;

MKP-1 reverse, 5′-TCCAGCATCCTTGATGGAGTCTATG-3′;

β-Actin forward, 5′-GCCACCAGTTCGCCATGGAT-3′;

β-Actin reverse, 5′-GCTTTGCACATGCCGGAGC-3′.

Statistical analysis of relative expression of the target gene based on comparative threshold values with efficiency correction was made with the Relative Expression Software tool (Bio-Rad CFX Manager Software), and was normalized to the housekeeping gene β-Actin. All experiments were repeated three times.

### Preparation of nuclear protein extracts

The nuclear extracts were prepared as described previously [28]. Cells were harvested and suspended in hypotonic buffer A (10 mM 4-(2-hydroxyethyl)-1-piperazineethanesulfonic acid (HEPES), pH 7.6, 10 mM KCl, 1 mM dithiothreitol (DTT), 0.1 mM EDTA, and 0.5 mM phenylmethylsulfonyl fluoride) for 10min on ice and vortexed for 10 s. Nuclei were pelleted by centrifugation at 12000 g for 20 s. The supernatants containing cytosolic proteins were collected. Nuclear pellet was suspended in buffer C (20 mM HEPES, pH 7.6, 1 mM EDTA, 1 mM DTT, 0.5 mM phenylmethylsulfonyl fluoride, 25% glycerol, and 0.4 M NaCl) for 30 min on ice. Nuclear protein containing supernatants were collected by centrifugation at 12000 g for 20 min and stored at −70°C.

### DNA affinity protein binding assay

After the indicated treatment, cells were harvested in buffer A, chilled on ice for 10 min and centrifuged at 12000 g for 20 s. Pellets were suspended in 5 times volume of buffer C and sonicated. A 200 µg aliquot of nuclear suspension was incubated with 2 µg of biotinylated double-stranded oligonucleotyde corresponding to the murine consensus NF-κB binding DNA sequence (Biotin-CCTTGAAGGGATTTCCCTCC, Invitrogen) for 30 min on a 4°C shaker-bath. Then, 30 µl streptavidin coated magnetic micro particles (Sigma-Aldrich) were added, and incubation was continued for an additional 30 min. Beads were pulled down, washed 3 times with ice-cold PBS, and eluted in 25 µl mercaptoethanol-free Laemmli sample buffer by a 5 min boiling. Eluted samples were subjected to immunoblot analysis. All experiments were repeated three times.

### In vitro kinase assay

RAW 264.7 macrophages were exposed to 1 µg/ml LPS for 1 h, washed in PBS and harvested in ice-cold lysis buffer containing 0,5 mM sodium metavanadate, 1mM EDTA, protease inhibitor mixture and 20 mM HEPES, pH: 7.4. Cell lysates were subjected to overnight immunoprecipitation at 4°C with anti-p38 MAPK, anti-JNK anti-ERK_1/2_ or anti-Akt antibodies. Precipitates were collected on appropriate secondary antibody-coated magnetic micro particles (Sigma-Aldrich) for 30 min. Beads were pulled down, washed 3 times with ice-cold PBS, and incubated for 10 min at 30°C in the presence or absence of 50 µM malvidin in 50 µl of buffer containing 25 mM glycerophosphate (pH 7.3), 0.5 mM dithiothreitol, 1.25 mM EGTA, 0.5 mM Na_3_VO_4_, 10 mM MgCl_2_, 1 mg/ml bovine serum albumin, 1 µM okadaic acid, 0.1 mM [γ-^32^P]ATP (250000 Bq/nmol; GE Healthcare Hungary Ltd, Budapest, Hungary) and 50 µg of recombinant Mef-2, c-Jun, MBP or GSK-3β protein. After incubation, aliquots were spotted on p81 filter paper, washed and counted for ^32^P radioactivity. Blank values were obtained by substituting a non-immune antibody preparation for the immunoprecipitating antibodies. All experiments were repeated three times.

### Statistical analysis

Each experiment was repeated at least three times. Values in the figures and text are expressed as mean ± S.E.M. of n observations. Statistical analysis was performed by analysis of variance followed by Student’s t-test. Statistical significance was set at p<0.05. For determining IC_50_ values from dose-response curves, the four-parameter logistic function of GraphPad Prism software was used.

## Results

### Malvidin inhibited LPS-induced NF-κB activation in RAW 264.7 macrophages

**Figure 1:**
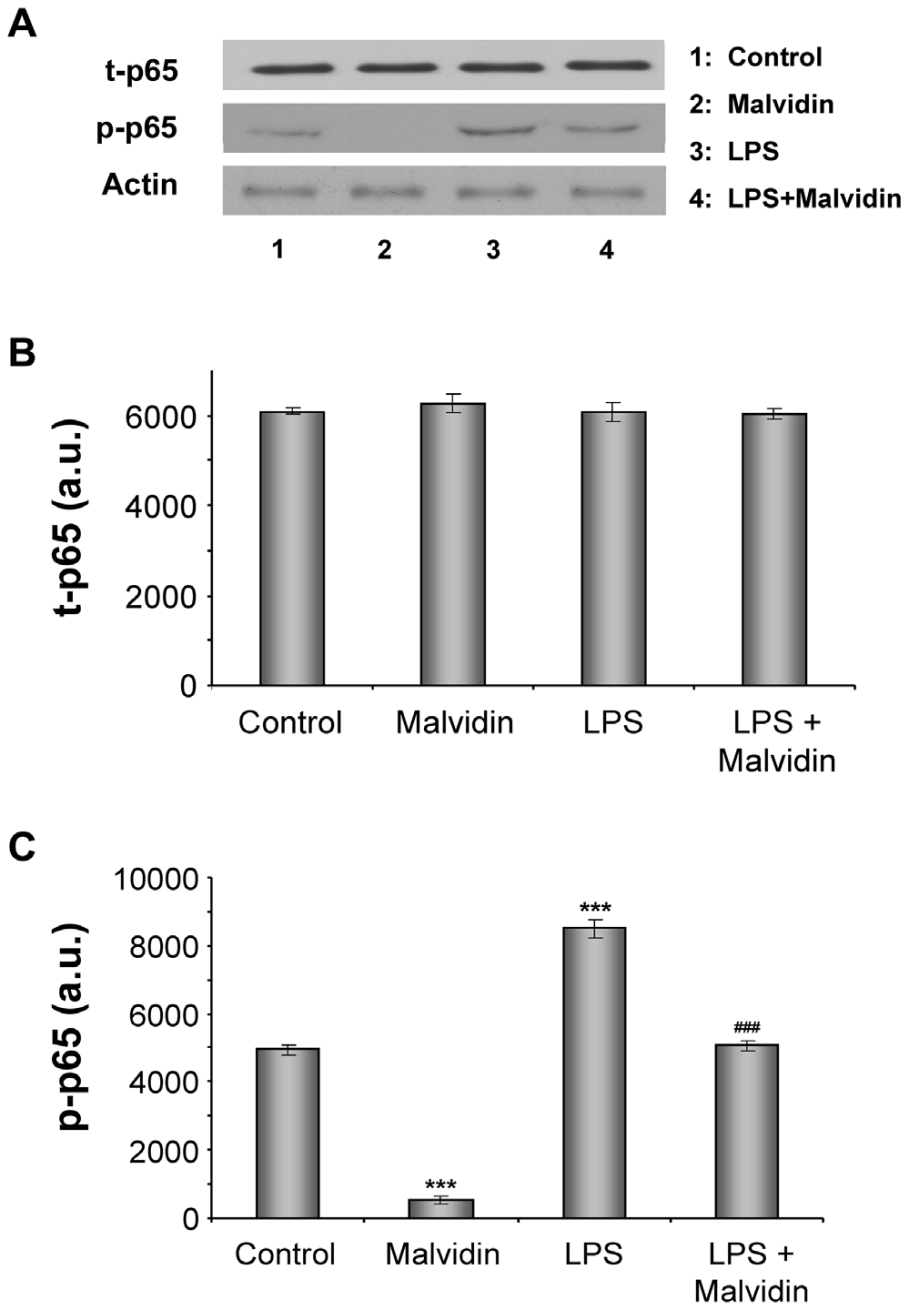
Effect of malvidin on LPS induced activating phosphorylation of NFκB. Total (phosphorylated and unphosphorylated) NFκB (t- NFκB) as well as the phoshorylated form of its p65 subunit (p-NFκB) was detected by immunoblotting of whole RAW 264.7 macrophage lysates after treating the cells for 1h as indicated. Actin was used as a loading control. Representative blots (A) and densitometric evaluations (B,C) of 3 independent experiments are shown. Pixel densities were normalized to that of the actin. Values are given as means ± SEM. *** p<0.001 compared to untreated control, ^###^ p<0.001 compared to LPS alone.

Phosphorylation of NF-κB p65 on Ser^536^ upon LPS stimulation enhances its transcriptional activity [29]. Therefore, we investigated whether malvidin affects LPS induced NF-κB p65 phosphorylation. To this end, we pre-incubated RAW 264.7 macrophages in the presence or absence of 50 µM malvidin for 30 min, then exposed or not the cells to 1 µg/ml LPS for 1 h, and determined steady state protein level and phosphorylation state of NF-κB by immunoblotting from whole cell homogenates. As demonstrated in Figure 1, LPS did not affect expression of NFκB, but induced phosphorylation of its p65 subunit. Malvidin effectively attenuated NF-κB phosphorylation both in the unstimulated and the LPS treated cells while did not affect expression of the protein (Fig. 1).

**Figure 2:**
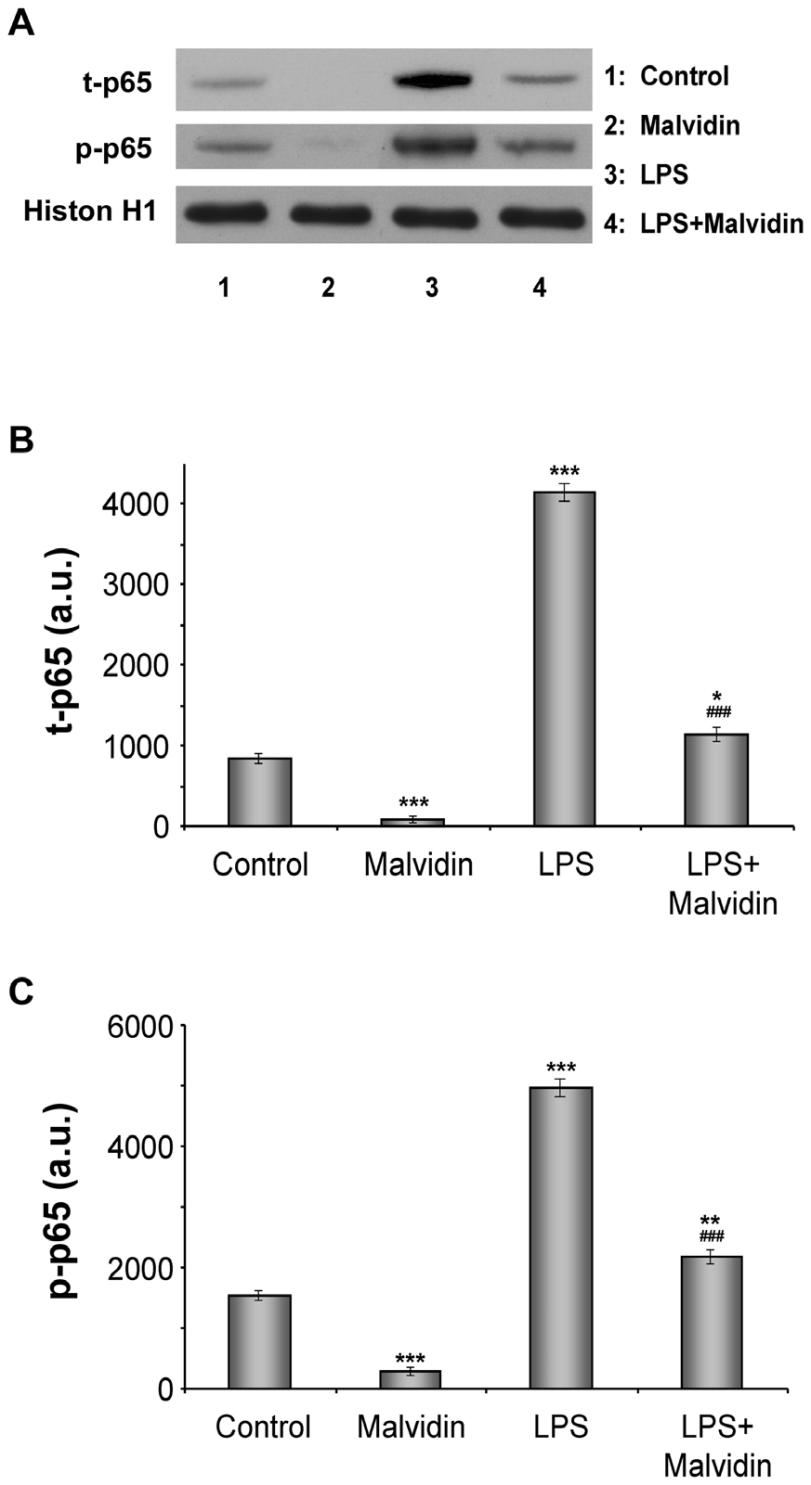
Effect of malvidin on LPS induced nuclear translocation and DNA binding of of NFκB. RAW 264.7 macrophages were treated for 1h as indicated, then nuclei were isolated and NFκB was extracted by using magnetic beads baited with oligonucleotides of the NFκB binding consesus sequence. Total (phosphorylated and unphosphorylated) NFκB (t-p65) as well as the phoshorylated form of its p65 subunit (p-p65) was detected by immunoblotting in the samples eluted from the beads. Histon H1 from the isolated nuclei was used as loading control. Representative blots (A) and densitometric evaluations (B,C) of three independent experiments are shown. Pixel densities were normalised to that of the histon H1. Values are given as means ± SEM. * p<0.05, ** p<0.01, *** p<0.001 compared to untreated control, ^###^ p<0.001 compared to LPS alone. a.u.: arbitrary units.

Activation of NF-κB presumes its translocation to the nucleus and its binding to the DNA. To determine nuclear translocation and DNA binding of NFκB, we isolated and homogenized nuclei of RAW 264.7 macrophages subjected to the aforementioned treatment protocol, and pulled down nuclear proteins by magnetic beads using oligonucleotides of the consensus NF-κB binding sequence as bait. Proteins eluted from the beads were subjected to immunoblot analysis. Figure 2 demonstrates LPS induced nuclear translocation and DNA binding of NF-κB. Malvidin attenuated these processes in unstimulated and LPS treated cells. Furthermore, we found the same pattern of alterations in total (phosphorylated and un-phosphorylated) and phosphorylated NF-κB (Fig. 2). This indicates that most of the nuclearly translocated NF-κBs were phosphorylated.

**Figure 3:**
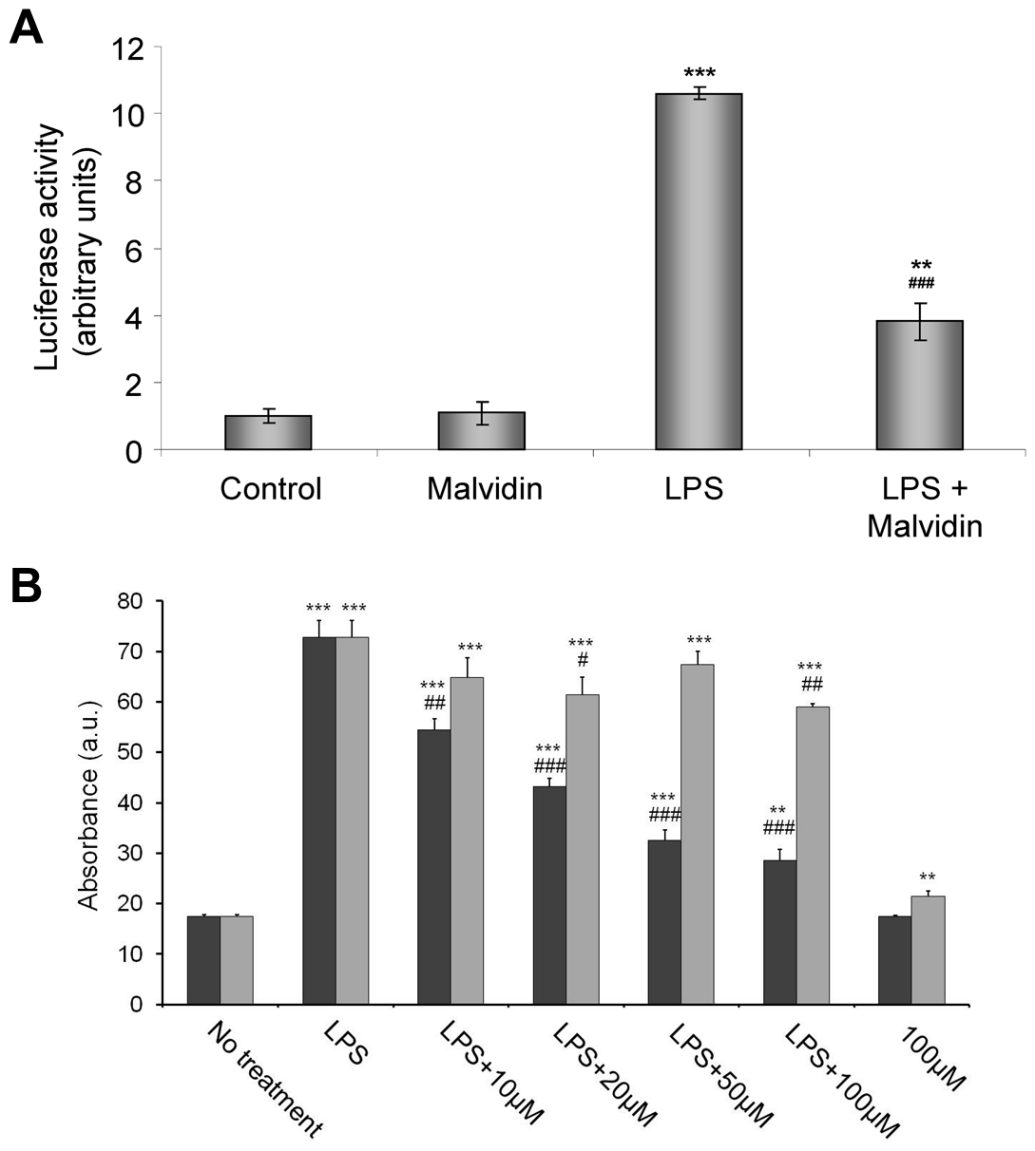
Effect of malvidin on LPS induced activation of NFκB in RAW 264.7 macrophages. Cells were pretreated with 0–100 µM malvidin (black bars in B) or 0–100 µM trans-resveratrol (gray bars in B) for 30 min as indicated. Activation of NF-κB was assessed by a luciferase (A) or an alkaline phosphatase (B) reporter assay after 1 µg/mL LPS exposion for 24 h. Values are given as means ± SEM of 4 independent experiments running in 3 parallels. ** p<0.01, *** p<0.001 compared to untreated control, ^#^ p<0.05, ^##^ p<0.01, ^###^ p<0.001 compared to LPS alone. a.u.: arbitrary units.

We confirmed the effect of malvidin on LPS induced NF-κB activation using functional luciferase reporter assay. We transiently transfected RAW 264.7 macrophages with NF-κB promoter driven luciferase constructDue to technical reasons, we treated the cells for 24 h instead of 1 before determining luciferase activity using chemiluminescence assay. We normalized our assay by co-transfecting the cells with a β-galactosidase expressing plasmid. Similarly to our previous two experiments, we found LPS induced the activation of NF-κB was attenuated by malvidin (Fig. 3A). In this assay, malvidin failed to decrease NF-κB activation in unstimulated cells (Fig. 3A).

We intended to compare NF-κB activation reducing effect of malvidin with that of trans-resveratrol. To this end, we treated RAW-Blue™ cells in the presence or absence of 0-100 µM malvidin or resveratrol with 1 µg/ml LPS for 24 h. RAW-Blue™ cells were permanently transfected with an NF-κB- and AP-1-sensitive promoter-driven alkaline phosphatase. Upon nuclear translocation, NF-κB and AP-1 binds to the promoter, and induces the expression of alkaline phosphatase. Alkaline phosphatase activity proportional to NF-κB activation was detected using a colour-changing subtrate containing assay medium. Malvidin inhibited LPS-induced NF-κB activation with the apparent IC_50_ value of 18.1±3.2 µM. Trans-resveratrol failed to inhibit NF-κB activation even at the highest concentration used (Fig. 3B).

### Malvidin inhibited LPS induced ROS production and PARP activation in RAW 264.7 macrophages

**Figure 4:**
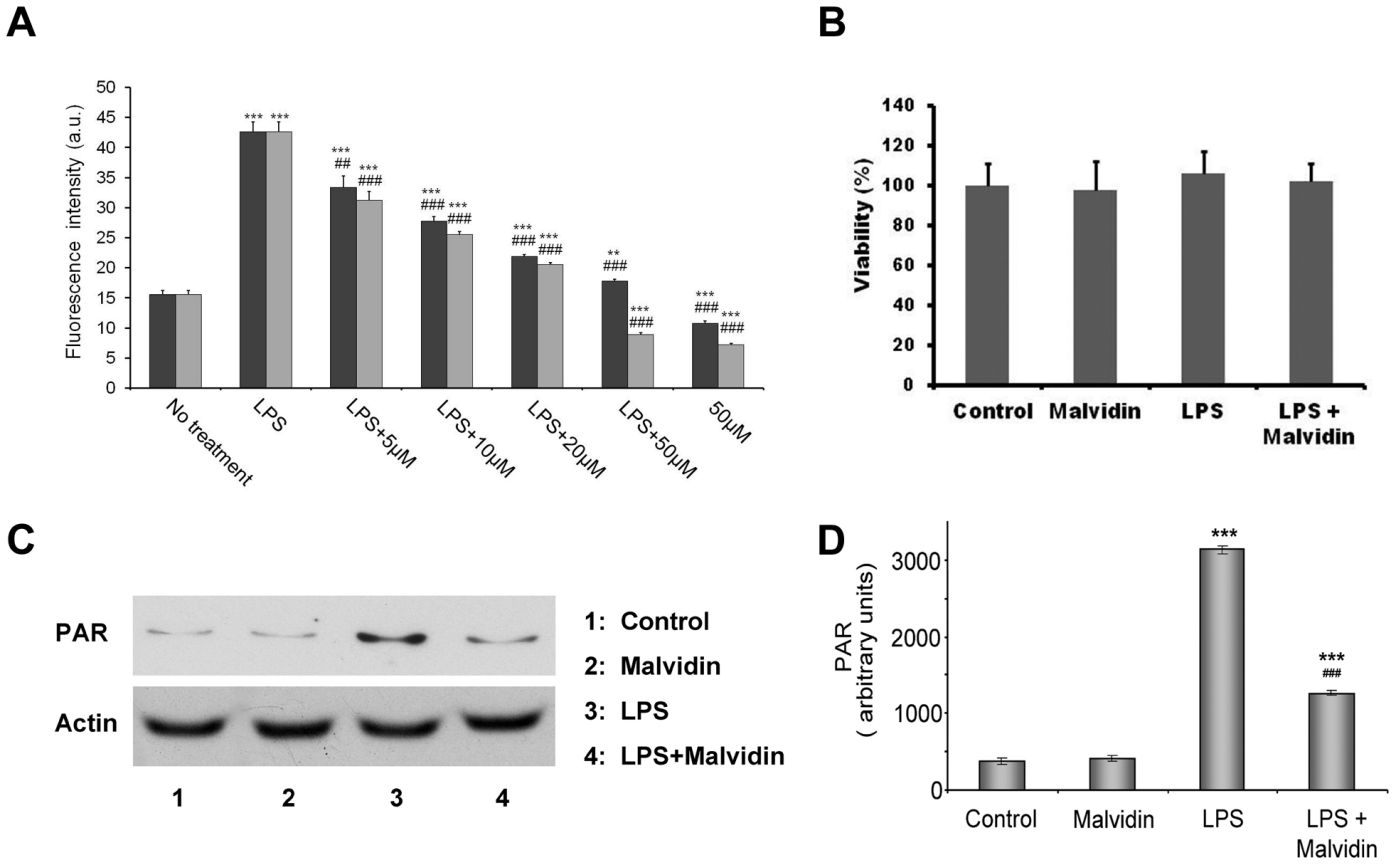
Effect of malvidin on LPS induced ROS production and PARP activation in RAW 264.7 macrophages. Steady state ROS concentration in the culturing medium (A) and viability of the cells (B) was determined using the fluorescent redox dye C-400 and by the MTT method, respectively after incubating the cells for 24 h in the absence and presence of LPS together with 0–50 µM malvidin (black bars in A) or trans-resveratrol (gray bars in A) as indicated. Experiments running in 6 parallels were repeated 3 times. PARP activation was assessed by determining the steady state level of PAR using immunoblotting from whole cell lysate after treating the cells for 1h as indicated. Actin was used as loading control. Representative blots (C) and densitometric evaluations (D) of three independent experiments are shown. Pixel densities were normalized to that of the actin. Values are given as means ± SEM. ** p<0.01 *** p<0.001 compared to untreated control, ^##^ p<0.01, ^###^ p<0.001 compared to LPS alone. a.u.: arbitrary units.

LPS is a well documented inducer of ROS production [30]. Therefore, we determined the effect of malvidin on ROS production in LPS-induced RAW macrophages using an oxidation sensitive fluorescent dye, C400. The cells were pre-incubated in the presence of 0–50 µM malvidin for 30 min, then exposed or not to 1 µg/ml LPS for 22 h. This was followed by additional 2 h incubation after supplementing the media with C400 at a final concentration of 2 µg/ml. Concentration of fluorescent C400 oxidized by the ROS was determined using fluorescence plate reader. Malvidin inhibited LPS induced ROS production in a concentration-dependent manner (Fig. 4A). We compared the antioxidant effect of malvidin with that of trans-resveratrol, and found it to be comparable (Fig. 4A). Apparent IC_50_ values for the two polyphenols were 9.0±0.8 and 6.8±0.6 µM, respectively. We investigated whether decreased ROS production was due to cytotoxic effect of malvidin by using MTT assay. Wefound cell viability was not affected by 50 µM malvidin during the 24 h incubation period (Fig. 4B).

LPS activates PARP in RAW 264.7 macrophages as the consequence of increased intracellular ROS induced breaks of one or both strands of the DNA [13]. Therefore, we investigated the influence of malvidin on LPS induced activation of PARP. We exposed the cells to the aforementioned treatment protocol, then assessed PARP activation by immunoblot analysis of the steady state level of the enzymatic product, PAR from whole cell homogenates. We found LPS induced PAR accumulation was attenuated by malvidin (Fig. 4C, D). Malvidin did not affect PARP activation in unstimulated cells (Fig. 4C, D).

### Malvidin inhibited LPS-induced MAPK activation in RAW 264.7 macrophages

**Figure 5:**
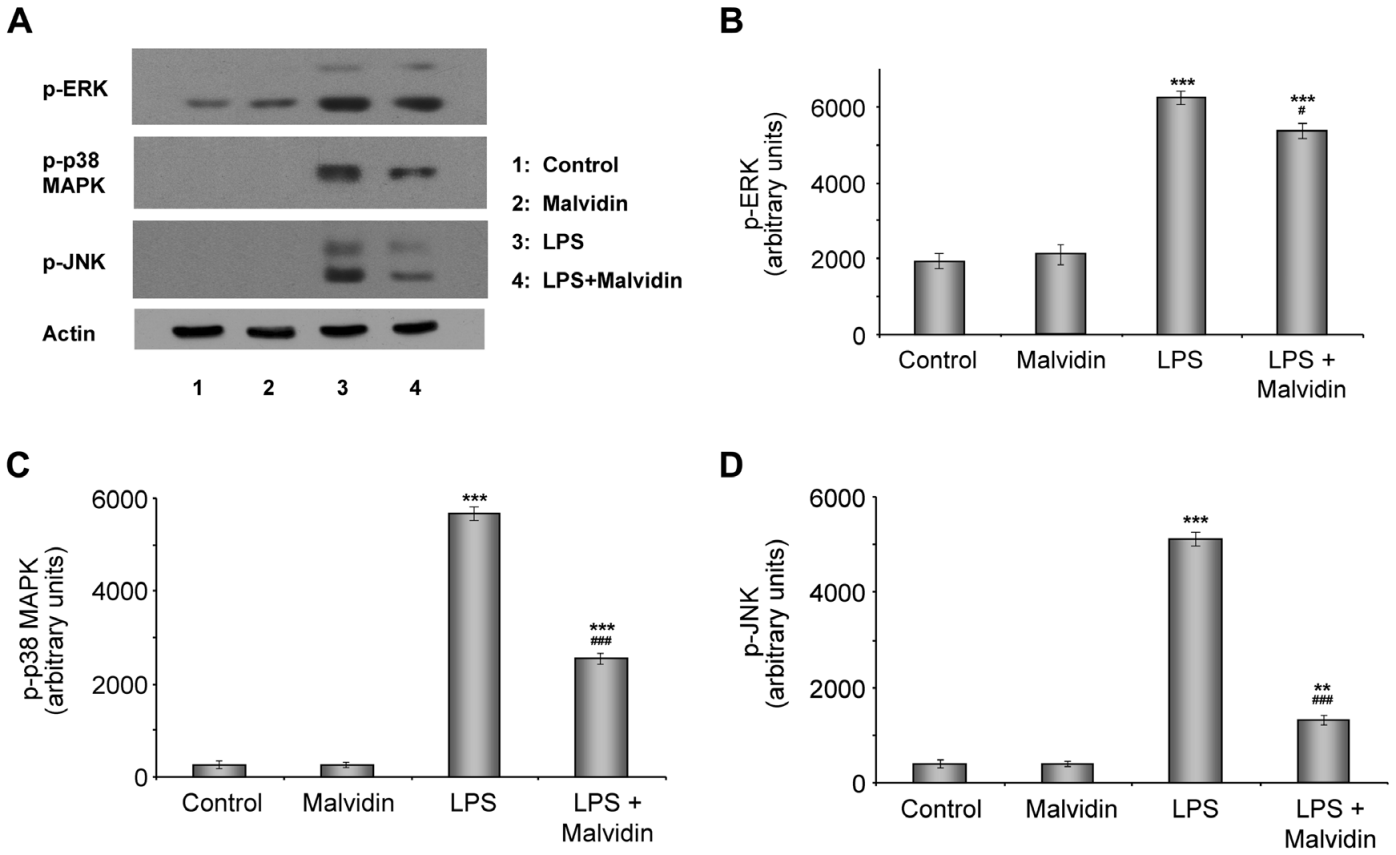
Effect of malvidin on LPS induced activation of ERK, p38, JNK MAPK in RAW 264.7 macrophages. Steady state phosphorylation of ERK, p38 and JNK was detected by immunoblotting from whole cell lysate after treating the cells as indicated for 1h. Actin was used as a loading control. Representative blots (A) and densitometric evaluations (B–D) of 3 independent experiments are shown. Pixel densities were normalized to that of the actin. Values are given as means ± SEM. ** p<0.01, *** p<0.001 compared to untreated control, ^#^ p<0.05, ^###^ p<0.001 compared to LPS alone.

Binding of LPS to the TLR4 receptor activates multiple intracellular signaling pathways including the MAPKs [31]. Therefore, we investigated the influence of malvidin on LPS induced activation of ERK, JNK and p38-MAPK. We preincubated or not RAW 264.7 macrophages with 50 µM malvidin for 30 min then treated them or not with 1 µg/ml LPS for 1 h. We performed immunoblot analysis utilizing phosphorylation specific primary antibodies from whole cell homogenates. Phosphorylation and thereby activation of the studied MAPKs were increased by LPS, which was attenuated by malvidin (Fig.5). This effect of malvidin was the least effective in the case of ERK_1/2_ (Fig. 5A, B), much more pronounced for p38 (Fig 5A, C) and the strongest for JNK (Fig.5 A, D). Malvidin did not exert any effect on the phosphorylation of MAPKs in unstimulated cells (Fig. 5).

### Malvidin enhanced MAPK phosphatase-1 (MKP-1) expression in unstimulated and LPS treated RAW 264.7 macrophages

**Figure 6:**
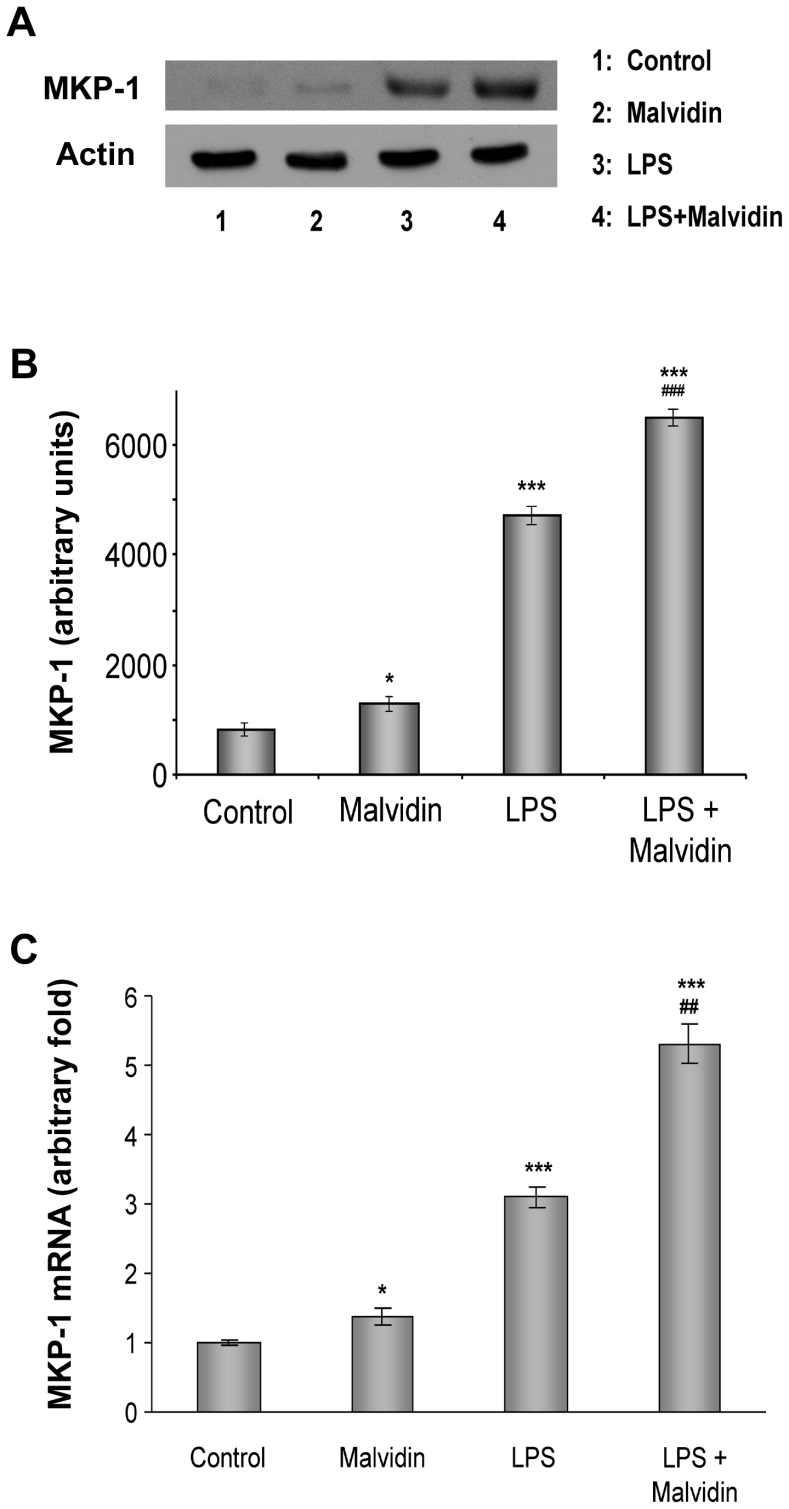
Effect of LPS and malvidin on MKP-1 expression in LPS treated RAW 264.7 macrophages. Effect of LPS and malvidin on steady state MKP-1 protein level was assessed by immunoblotting from whole cell lysate after treating the cells as indicated for 1h. Actin was used as a loading control. Representative blots (A) and densitometric evaluations (B) of 3 independent experiments are shown. Pixel densities were normalized to that of the actin. MKP-1 mRNA expression (C) was determined in another aliquot of cells treated as above using Q-RT-PCR analysis. β-Actin was used as a housekeeping control gene. Specific primer sequences and PCR conditions are described in Materials and Methods. Values are given as means ± SEM. * p<0.05, *** p<0.001 compared to untreated control, ^##^ p<0.01 ^###^ p<0.001 compared to LPS alone.

MKP-1 dephosphorylates thereby down-regulates the activity of all three branches of MAPKs [32]. Therefore, we determined how malvidin affects MKP-1 expression in unstimulated and LPS treated RAW 264.7 macrophages. We subjected the cells to the aforementioned treatment protocol, and performed immunoblot analysis from whole cell homogenates. After mRNA isolation and cDNA transcription, we performed Q-RT-PCR amplification assay. We found that LPS induced MKP-1 mRNA (Fig. 6C) and protein (Fig. 6A, B) expression. Malvidin increased MKP-1 expression in unstimulated cells in a much lower extent than LPS. In LPS stimulated cells, malvidin increased MKP-1 mRNA and protein much above the level that of LPS alone (Fig. 6).

### Malvidin enhanced PI-3-kinase-Akt pathway activation in unstimulated and LPS treated RAW 264.7 macrophages

**Figure 7:**
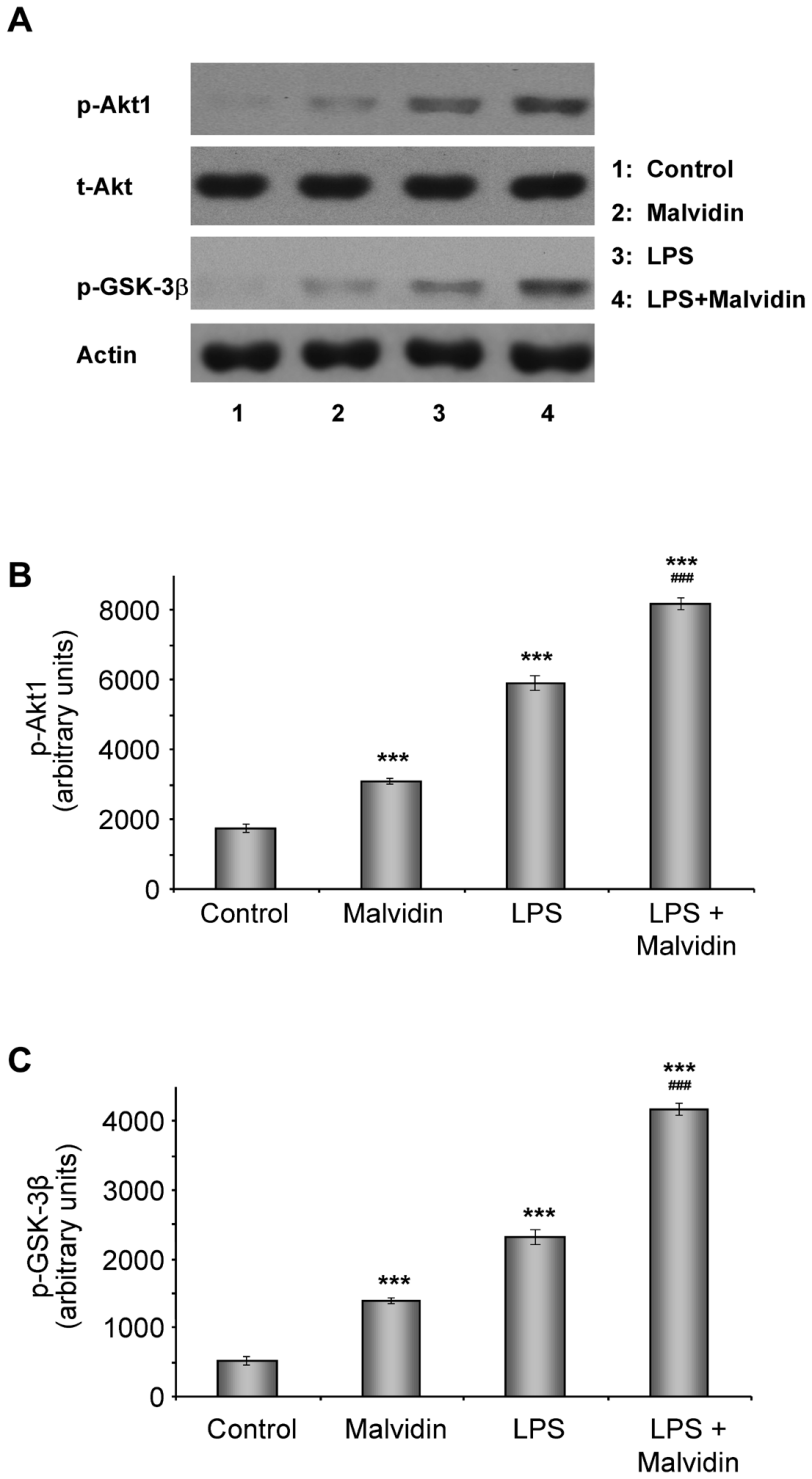
Effect of malvidin on LPS induced activation of Akt1 in RAW 264.7 macrophages. Steady state level of total (phosphorylated and unphosphorylated) Akt1 (t-Akt) as well as phosphorylation of Akt1 and its down-stream target GSK-3β was detected by immunoblotting from whole cell lysate after treating the cells as indicated for 1h. Actin was used as loading control. Representative blots (A) and densitometric evaluations (B–C) of 3 independent experiments are shown. Pixel densities were normalized to actin. Values are given as means ± SEM. *** p<0.001 compared to untreated control, ^###^ p<0.001 compared to LPS alone.

It was previously shown polyphenols modulate the phosphatidylinositol 3 (PI3)-Kinase-Akt pathway [33]. Furthermore, we previously found that activation of this cytoprotective pathway was a beneficial factor of PARP inhibition in a murine endotoxic shock model [34]. Therefore, we investigated the effect of malvidin on the phosphorylation of Akt and its down-stream target, GSK-3ß in unstimulated and LPS treated RAW 264.7 macrophages using immunoblot analysis. We followed the same experimental protocol as we did for MAPK activation studies. We found LPS increased activation of Akt as it was revealed by the phosphorylation of its Ser^473^ and GSK-3ß (Fig. 7). Malvidin increased Akt (Fig. 7A, B) and GSK-3ß (Fig. 7A, C) phosphorylation in unstimulated cells in a lower extent than LPS. In LPS stimulated cells, malvidin increased phosphorylation of both proteins much above the level that of LPS alone (Fig. 7).

### Malvidin protected mitochondrial membrane potential from LPS induced depolarization in RAW264.7 macrophages

**Figure 8:**
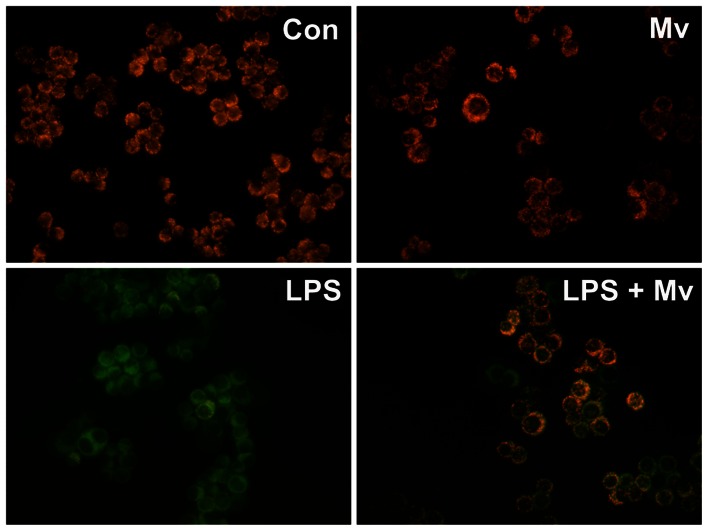
Effect of LPS and malvidin on mitochondrial membrane potential of RAW 264.7 macrophages. Cells were pretreated or not with malvidin for 30 min and exposed or not to LPS for 1h. Medium was replaced to fresh one without any agents and containing 1 µg/ml JC-1 membrane potential-sensitive fluorescent dye for 15 min. Green and red fluorescence images of the same field were acquired using a fluorescent microscope. Representative merged images of three independent experiments are presented. Con: control; Mv: malvidin.

Increased ROS and MAPK activation damages while Akt activation protects integrity of the mitochondrial membrane systems [35]. To investigate the impact of LPS and malvidin on mitochondrial membrane potential, we used a cell-permeable voltage-sensitive fluorescent mitochondrial dye, JC-1, and fluorescent microscopy. After treating them according to the aforementioned protocol, we loaded the cells with JC-1 for 15 min, and acquired fluorescence images of the same area of interest in the green and red channels of the microscope. Mitochondrial membrane depolarization was indicated by the disappearance of the red component of JC-1 fluorescence while normal membrane potential was demonstrated by balanced red and green color. Figure 8 clearly demonstrates that LPS caused significant mitochondrial membrane depolarization that was attenuated by malvidin. Malvidin did not exert any effect on mitochondrial membrane integrity in unstimulated cells (Fig. 8).

### Malvidin, kinase inhibitors and N-acetyl cysteine (NAC) attenuate nuclear translocation and DNA binding of NF-κB in different extent

**Figure 9:**
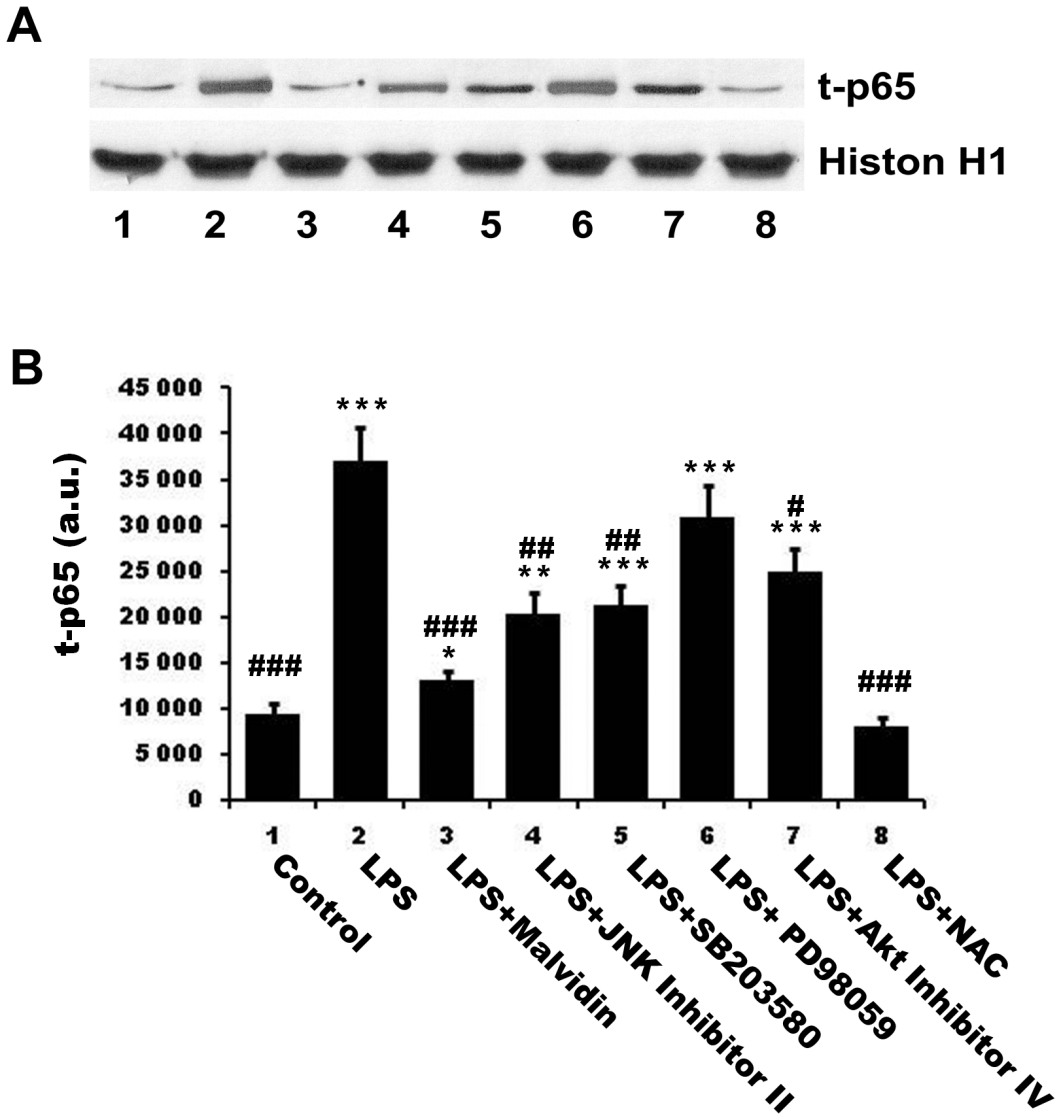
Effect of malvidin, kinase inhibitors and NAC on LPS induced nuclear translocation and DNA binding of of NFκB. RAW 264.7 macrophages were treated for 1h as indicated, then nuclei were isolated and NFκB was extracted using magnetic beads baited with oligonucleotides of NFκB binding consesus sequence. Total (phosphorylated and unphosphorylated) NFκB (t-p65) was detected by immunoblotting in the samples eluted from the beads. Histon H1 from the isolated nuclei was used as loading control. Representative blots (A) and densitometric evaluations (B) of 3 independent experiments are shown. Pixel densities were normalized to histon H1. Values are given as means ± SEM. * p<0.05, ** p<0.01, *** p<0.001 compared to untreated control, ^#^ p<0.05, ^##^ p<0.01, ^###^ p<0.001 compared to LPS alone. a.u.: arbitrary units; SB203580: p38 MAPK inhibitor; PD98059: ERK inhibitor; NAC: N-acetyl cysteine.

To establish the physiological significance of malvidin’s effects on signalling pathways, we compared its effect with that of various kinase inhibitors and the ROS scavenger NAC on nuclear translocation and DNA binding of NFκB. To this end, we preincubated or not RAW 264.7 macrophages with 50 µM malvidin, 1 µM JNK Inhibitor II, 1 µM SB203580 (p38 inhibitor), 25 µM PD98059 (ERK inhibitor), 5 µM Akt Inhibitor IV or 3 mM NAC before 1 h exposure to 1 mg/ml LPS. We isolated and homogenized nuclei of the cells subjected to the aforementioned treatment protocol, and pulled down nuclear proteins by magnetic beads using oligonucleotides of the consensus NF-κB binding sequence as bait. Proteins eluted from the beads were subjected to immunoblot analysis utilizing anti-p65 primary antibody. We found NAC abolished LPS induced nuclear translocation and DNA binding of NF-κB. The other substances but ERK inhibitor attenuated NF-κB activation in different extent (malvidin >JNK∼p38>Akt inhibitor, Fig. 9). ERK inhibition also diminished NF-κB activation, however, it did not reach the threshold of statistical significance.

Malvidin was identified as a rather potent inhibitor of cAMP phosphodiestherase (IC_50_ 23±5 µM), thereby a potential indirect regulator of MAPKs [36]. We performed *in vitro* radioactive kinase assays utilizing enzymes immunoprecipitated from lysate of LPS activated RAW 264.7 macrophages and recombinant substrates to determine whether malvidin had any direct effect on the kinases studied. We found malvidin did not exert any direct effect on the MAPKs or Akt up to 50 µM concentration (data not shown).

## Discussion

**Figure 10:**
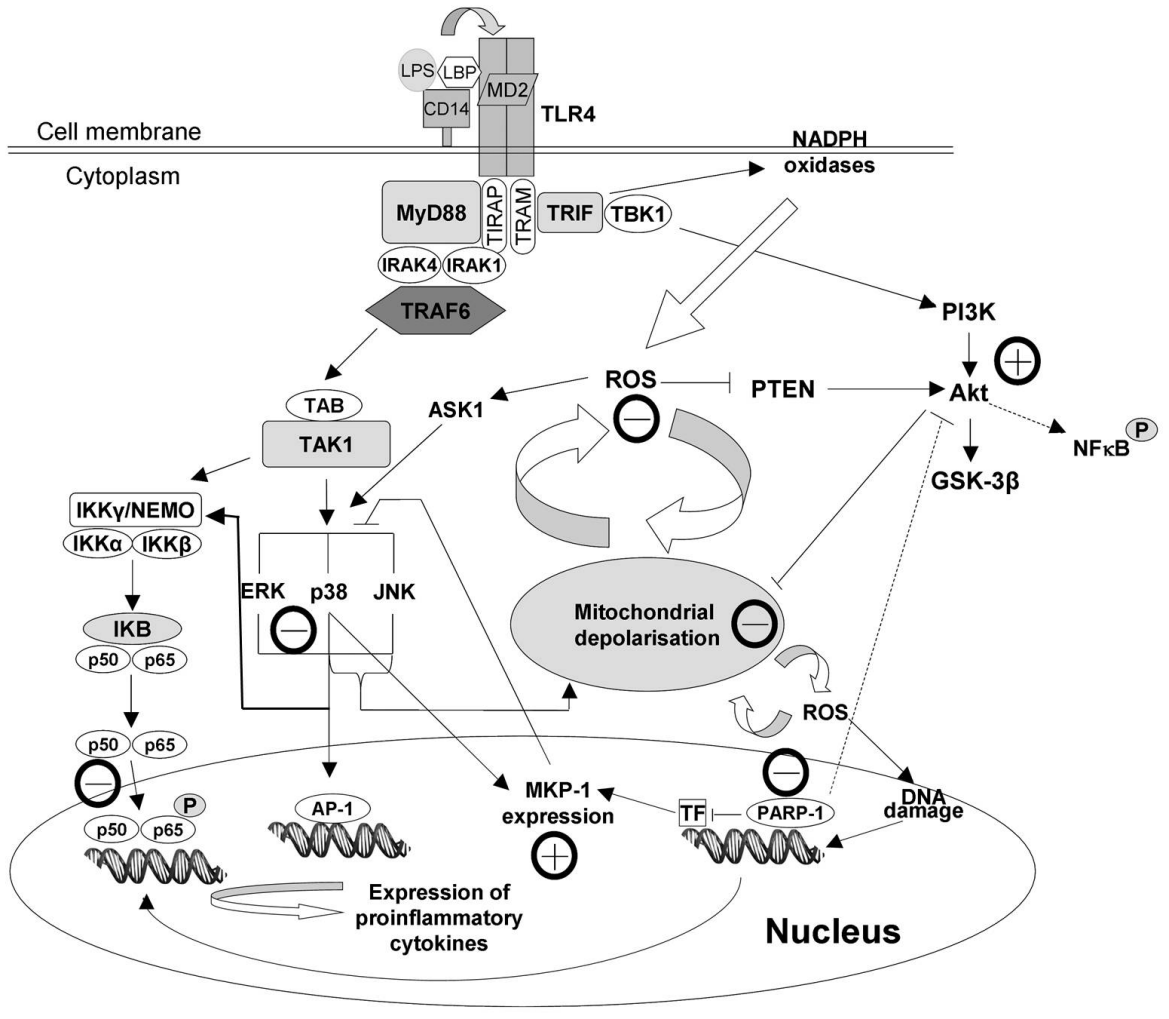
Effect of malvidin on LPS induced pathophysiological changes in RAW 264.7 macrophages. Well documented effects are indicated by solid lines, whereas effects involving yet unidentified mediator(s) or events are represented by dashed line. Lines with pointed end denote activation, whereas lines with a flat end indicate inhibition. Activating or inhibitory effect of malvidin is indicated by a circled + or − next to the line, respectively. LPS induces activating phosphorylation, nuclear translocation and DNA binding of NFκB, induction of ROS production, PARP activation, activation of MAPKs, MKP-1 expression, activation of the phosphatidylinositol-3 kinase-Akt pathway and destabilization of the mitochondrial membrane systems. Malvidin attenuates ROS production, mitochondrial destabilization, and activation of PARP and MAPKs. It also augments Akt activation and MKP-1 expression resulting in diminished activation of NFκB. AP-1: activator protein-1; Akt: Protein Kinase B (PKB); Ask1: apoptotic signal regulating kinase; ERK: extracellular signal-regulated kinase; GSK-3β: Glycogen synthase kinase 3 beta; IκB: inhibitors of NF-κB; IKK: inhibitor of NF-kappa B kinase; IRAK1: interleukin-1 receptor-associated kinase-1; IRAK4: interleukin-1 receptor-associated kinase-4; JNK: c-jun N-terminal kinase; LBP: LPS binding protein; LPS: lipopolysaccharide; MKP-1: MAPK phosphatase -1; MyD88: myeloid differentiation primary response gene 88; NEMO: NF-κB essential modifier; NF-kappa B: nuclear factor kappa B; P: phosphorylated; PARP: poly-(ADP-ribose) polymerase; p65: Transcription factor p65 (RelA); p50: NF-KappaB1; PI3K: phosphoinositide 3-kinase; PTEN: phosphatase and tension homolog deleted on chromosome 10; ROS: reactive oxygen species; TAB: TAK1-binding protein; TAK1: transforming growth factor- β-activated kinase 1; TBK1: TANK binding kinase 1; TF: transcription factor; TIR: Toll-interleukin-1 receptor; TIRAP: TIR domain-containing adaptor protein; TLR4: Toll-like receptor 4; TRAF6: TNF receptor-associated factor 6; TRAM: TRIF-related adaptor molecule; TRIF: TIR domain-containing adaptor inducing IFN-β.

In response to LPS, nuclear localization signal of cytosolic NF-κB becomes unmasked resulting in nuclear translocation of the transcription factor. In the nucleus, NF-κB becomes phosphorylated and acetylated, thus activated to bind to its consensus promoter DNA sequences. This binding triggers the expression of its target genes (Fig. 10) including pro-inflammatory cytokines, chemokines, adhesion proteins, COX-2 and iNOS [10], [37], [38]. These events are of pivotal importance in the development of inflammation-related chronic diseases [39]. We demonstrated malvidin attenuates activating phosphorylation, nuclear translocation and binding to consensus DNA sequence of NF-κB. These data are completely in line with the results of other groups [8], [26], [40]. Furthermore, we found malvidin antagonised NF-κB activation at much lower concentrations than trans-resveratrol. This indicates malvidin could account for the beneficial effects of red wine in inflammation-related chronic diseases. Furthermore, these results explain the finding of the 1999–2002 US National Health and Nutrition Examination Survey describing malvidin intake negatively correlates with serum C-reactive protein levels [25].

Binding of LPS to TLR4 receptor triggers activation of the MAPKs (Fig. 10) via various signaling pathways such as the myeloid differentiation primary response gene (MyD)88—interleukin-1 receptor-associated kinase (IRAK)—tumor necrosis factor (TRAF)-6—transforming growth factor-β activated kinase (TAK) pathway [32]. In turn, MAPK pathways are involved in activation of the pro-inflammatory transcription factors; NF-κB and AP-1 [32], [37]. In the present study, we observed malvidin attenuated LPS induced activation of all three MAPKs. However, this effect differed for the three kinases (JNK>p38>>ERK). By using specific kinase inhibitors, we aimed to establish the significance of these results. In agreement with others [41]-[43] we found JNK and p38 inhibitors significantly reduce LPS induced nuclear translocation and DNA binding of NF-κB. However, ERK inhibition was ineffective. These data indicate early inflammatory response in RAW 264.7 macrophages is mediated, at least partially, via the aforementioned pathway. On the other hand, it is likely that malvidin decreased LPS evoked MAPK activation undirectly since in *in vitro* kinase assays malvidin did not exert any effect. Most likely, it regulated MAPK activation by inhibiting other key mechanisms; ROS production.

MKP-1 is the major enzyme responsible for the dephosphorylation, thereby inactivation of all three MAPKs [44]. It is critically involved in inflammatory signaling of macrophages, and is responsible for switching off pro-inflammatory cytokine production *in vitro* and *in vivo* [41], [42]. In agreement with others [44] we found increased expression of MKP-1 in the LPS stimulated macrophages both at the mRNA and protein level. However, this was accompanied by an elevated activation of the MAPKs indicating MKP-1 induction was not sufficient to suppress LPS-induced MAPK activation. Malvidin enhanced MKP-1 expression both in the unstimulated and LPS treated cells, which was accompanied by decreased activation of the MAPKs. This suggests MKP-1 expression, when augmented by malvidin, could counteract the activating mechanisms induced by TLR4 signaling (Fig. 10). However, we found significant differences among the MAPKs regarding malvidin’s effectivity in reduction of their LPS induced activation. Furthermore, these differences were reflected in the anti-inflammatory effect of MAPK inhibitors. All these data indicate, the network of MAPK activation and inhibition signaling is complex, and balance of the regulating processes differs for each MAPK.

Previous studies established *in vitro* antioxidant characteristics for malvidin [3], [45]. In agreement with these results, we found malvidin attenuates ROS production by LPS-treated RAW264.7 macrophages at an IC_50_ value comparable to that of trans-resveratrol. At the same time, this modulated a complicated network of processes produced and regulated by ROS (Fig. 10) including mitochondrial integrity and activation of MAPKs, Akt and PARP. It is feasible that LPS induced NF-κB activation in our experimental system was mediated partially via the TLR4-NADPH oxidases-ROS-PARP pathway. However, the complexity of the involved networks made it hard to distinguish between cause and consequence or identify up-stream and down-stream events. Nevertheless, it is likely that due to its antioxidant property, malvidin decreases ROS production, thereby reduces PARP and MAPK activation as well as oxidative damage to MKP-1. Reduced PARP activation leads to decreased NF-κB and MAPK activation, increased expression of MKP-1 [15] and activation of the PI3K—Akt pathway [34] that together with the decreased ROS results in maintained mitochondrial integrity (Fig. 10). Importance of the antioxidant mechanism in malvidin’s anti-inflammatory effect is emphasized by us and others [46], [47] reporting NAC inhibits LPS induced NF-κB activation.

Recently it has been shown that Akt is a downstream target of TRIF/TANK-binding kinase 1 (TBK1), and there is an association between endogenous TBK1 and Akt in LPS treated macrophages. TBK1 enhances phosphorylation of Akt on Ser(473), and siRNA-mediated silencing or knocking out of TKB1 compromises LPS induced Akt activation [48]. On the other hand, elevated ROS also activates the PI-3K—Akt pathway via oxidative inactivation of the phosphatase and tensin homolog (PTEN) that inactivates the pathway by dephosphorylation [49]. Akt activation may result in mitochondrial protection by phosphorylation, thereby inactivation of Bad, and indirect NF-kappaB activation [50]. As we found, malvidin activated Akt both in the unstimulated and LPS-treated macrophages. Most likely, this effect of malvidin was also due to its antioxidant property. The augmented activation of Akt was most probably involved in malvidin’s protective effect on LPSinduced mitochondrial depolarization (Fig. 10).On the other hand, Akt was implicated in the phosphorylation thereby activation of NF-κB p65 [51]. Accordingly and in agreement with Zhao et al. [52], we found that inhibition of the PI-3K-Akt pathway attenuated NF-κB activation suggesting a partial involvement of this pathway in mediating LPS’s effect. All these data suggest Akt activating effect was unlikely to be involved in malvidin’s anti-inflammatory effect.

In conclusion, malvidin, the most abundant polyphenol ingredient of red wine, augments LPS-induced Akt activation and MKP-1 expression and attenuates mitochondrial destabilization, ROS production and activation of PARP as well as MAPKs resulting eventually in diminished activation of NFκB. All these data indicate malvidin significantly contributes to the antioxidant and anti-inflammatory effects of red wine, and could, at least partially, account for the positive effects of moderate red wine consumption on inflammation-mediated chronic maladies such as obesity, diabetes, hypertension and cardiovascular disease.
